# Platelet-Derived Growth Factor Subunit A Strengthens the Neurovascular Unit and Inhibits Retinal Vascular Regression Under Hyperoxic Conditions

**DOI:** 10.3390/ijms252312945

**Published:** 2024-12-02

**Authors:** Kaito Yokota, Haruhiko Yamada, Hidetsugu Mori, Yuki Hattori, Masatoshi Omi, Yuichi Yamamoto, Keiko Toyama, Hisanori Imai

**Affiliations:** Department of Ophthalmology, Kansai Medical University, 2-5-1 Shin-machi, Hirakata 573-1010, Osaka, Japan

**Keywords:** retinopathy of prematurity, astrocytes, hyperoxia, platelet-derived growth factor subunit A, astrocyte-vascular unit

## Abstract

Retinopathy of prematurity (ROP) is primarily caused by the exposure of preterm infants with underdeveloped blood vessels to high oxygen concentrations. This damages the astrocytes that promote normal vascular development, leading to avascularity, pathological neovascularization, and retinal detachment, and even blindness as the disease progresses. In this study, the aim was to investigate the differences in the characteristics of astrocytes and blood vessels between wild-type (WT) and genetically modified mice overexpressing platelet-derived growth factor subunit A (PDGF-A) in the retina immediately after high oxygen exposure, a protocol in the oxygen-induced retinopathy (OIR) model of ROP. Our results showed that PDGF-A mice exhibited an increased population of astrocytes and higher vascular density than WT mice and that PDGF-A strengthened the resistance to hyperoxic conditions. In the OIR model, PDGF-A mice had reduced avascular zone areas following hyperoxia exposure. Furthermore, immunostaining for NG2 and CD31 showed that pericytes tended to regress earlier than endothelial cells, particularly at the vessel edges in both WT and transgenic mice, indicating relatively higher susceptibility to hyperoxia-induced damage. These findings suggest that PDGF-A plays a crucial role in stabilizing retinal vessels and may serve as a novel therapeutic target for ROP, highlighting the potential significance of PDGF-A in the pathological mechanisms of retinal diseases.

## 1. Introduction

Retinopathy of prematurity (ROP), which is characterized by abnormal blood vessel growth in the retina, is a significant cause of visual impairment in premature infants. This condition primarily arises due to the premature interruption of normal retinal vascular development and subsequent exposure to varying oxygen levels in neonatal care units [1]. In severe cases, the abnormal proliferation and disruption of normal immature retinal blood vessel development can lead to retinal detachment and vision loss. Treatments such as retinal photocoagulation and intravitreal anti-vascular endothelial growth factor (VEGF) injections are effective in managing abnormal blood vessel growth [2], but no currently available treatments can truly treat ROP by restoring the retinal vascular networks that have already become avascular [1,2,3].

Oxygen-induced retinopathy (OIR) is a well-established animal model that closely replicates the pathogenesis of ROP [4]. In this model, neonatal mice are exposed to hyperoxia followed by a return to normoxia, and this exposure leads to retinal vaso-obliteration and subsequent pathological neovascularization, mirroring the phases of vessel loss and aberrant proliferation observed in ROP. However, unlike in humans, the non-perfused areas and the associated neovascularization in mice recover with time, eventually returning to an almost normal state [4].

Astrocytes are retinal glial cells that play a crucial role in the development and maintenance of retinal blood vessels [5]. During retinal development, astrocytes migrate from the optic nerve head to the peripheral retina and provide a scaffold for endothelial cells to form new blood vessels by secreting VEGF, which stimulates angiogenesis and promotes the formation of a stable vascular network [6]. Notably, this process is significantly influenced by oxygen levels. The astrocytes initially originate from precursor cells that express Pax2 under hypoxic conditions, which promote astrocyte migration and proliferation. As these precursor cells invade the retina, they proliferate rapidly and begin to express low levels of glial fibrillary acidic protein (GFAP) [6]. Postnatally, they differentiate into immature astrocytes, laying the foundation for the retinal vasculature. As the retina further matures and vascularizes, astrocytes guide and stabilize developing blood vessels, preventing pathological neovascularization. Mature astrocytes, marked by strong GFAP immunoreactivity, then participate in vessel pruning and remodeling in response to oxygen levels, ensuring proper vascular formation and maintenance [7].

Platelet-derived growth factors (PDGFs) are a family of growth factors that play a significant role in regulating cell proliferation, migration, and survival, particularly in the context of vascular and neural development. PDGFs consist of four polypeptide chains (PDGF-A, -B, -C, and -D) that form functional homodimers or heterodimers (e.g., PDGF-AA, -BB, -AB) and act through two tyrosine kinase receptors, PDGFR-α and PDGFR-β [8]. Each PDGF isoform exhibits distinct binding affinities and biological functions. For instance, PDGF-A and PDGF-B have distinct roles, with PDGF-A being crucial for astrocyte development and PDGF-B primarily involved in pericyte recruitment and vascular stabilization [8]. Together, these signaling pathways are essential for the development and maintenance of the retinal vasculature, influencing processes such as angiogenesis and vascular remodeling.

Studies have shown that PDGF-A regulates astrocyte activity by promoting their proliferation and differentiation, thereby supporting normal angiogenesis, and thus playing a crucial role in the development of retinal blood vessels [9]. PDGF-A signaling is essential for the survival and function of astrocytes, and its deficiency leads to the collapse of the retinal vascular network. Therefore, the balance between PDGF-A signaling, astrocyte activity, and vascular development is expected to be critical for the normal development of the retinal vascular network and potentially for the prevention of diseases characterized by vascular regression, such as ROP. Previous studies on transgenic mice overexpressing PDGF-A in the retina [10,11] have reported vascular regression following OIR and a reduction in tuft formation at postnatal day 17 (P17). However, the precise mechanisms underlying these phenomena have not been thoroughly explored.

In this study, we investigated the vascular regression following hyperoxic exposure in one of the abovementioned transgenic PDGF-A OIR models, focusing on the effects of PDGF-A on astrocytes and blood vessels and its role in astrocyte-mediated vascular stabilization. By comparing the transgenic and wild-type (WT) mice under the high-oxygen exposure OIR protocol as a model for ROP, we aimed to further understand the potential of PDGF-A as a therapeutic target for this condition.

## 2. Results

### 2.1. Elevated PDGF-A Expression in Transgenic Mice at P7

**Figure 1:**
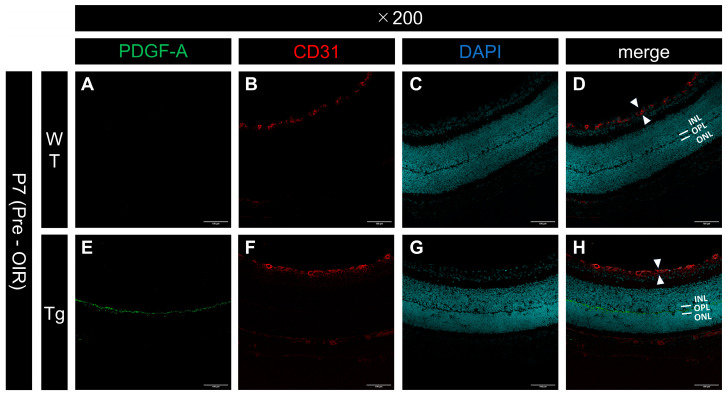
Confocal images of frozen sections of eyes from P7 WT and PDGF-Atg mice immunostained with anti-PDGF-A (green) and anti-CD31 (red) antibodies and DAPI (blue). (**A**–**D**) WT mouse retina; (**E**–**H**) retinas from genetically modified mice. Scale bars = 100 μm, 200×); (**A**,**E**) PDGF-A antibody staining; (**B**,**F**) CD31 staining; (**C**,**G**) DAPI; (**D**,**H**) merged images. No clear PDGF-A expression is observable in the WT retina (**A**,**D**), whereas PDGF-A expression is observed in the outer plexiform layer (OPL), which corresponds to the inner segment of the rhodopsin-producing photoreceptor, in genetically modified mice (**E**,**H**). In both wild-type (white arrow in (**D**)) and genetically modified (white arrow in (**H**)) mice, blood vessels (CD31) were observed only in the superficial layer of the retina, with no differences in the vascular layer structure.

**Figure 2:**
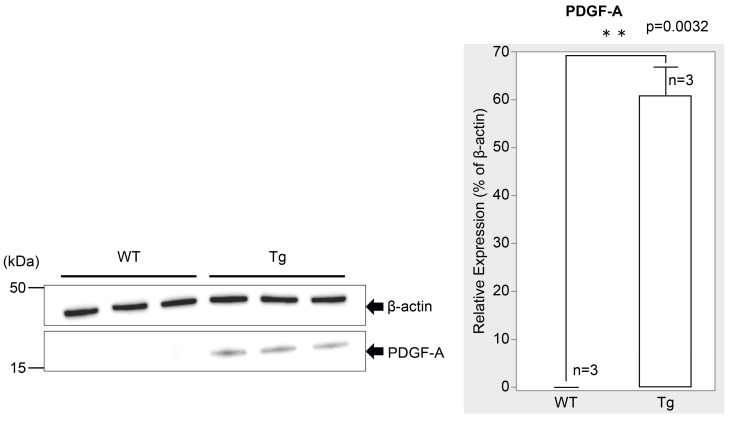
Western blot analysis of retinal tissue showing PDGF-A expression in WT (n = 3) and genetically modified (Tg; n = 3) mice at P7 (normalized to β-actin). WT retinas showed no detectable PDGF-A expression, while Tg retinas showed detectable PDGF-A expression (n = 3, *p* = 0.0032 < 0.05), indicating a significant difference between the groups. The bars represent the mean ± standard error of the mean (SEM) values.

PDGF-Atg mice had significantly higher PDGF-A expression than WT mice at P7. Immunofluorescence imaging revealed markedly higher PDGF-A staining intensity in transgenic retinas (Figure 1E,H) relative to WT retinas (Figure 1A,D), particularly in the outer plexiform layer (OPL). This increase was further confirmed by Western blotting analysis, which showed a significant increase in PDGF-A levels in transgenic mice (Figure 2; n = 3, *p* = 0.0032), with quantitative analysis underscoring the substantial upregulation of PDGF-A expression due to the genetic modification in transgenic mice compared to WT controls.

### 2.2. No Differences in Vascular Layer Structure in Transgenic Mice at P7

At P7, the vascular layer is observed only in the superficial layer of the retina [3]. Immunostaining of frozen sections for vascular endothelial cells (CD31+) was used to confirm that the vascular layer was restricted to the superficial layer in both the WT (Figure 1B,D) and PDGF-Atg (Figure 1F,H) mice, with no discernible differences between the two groups.

### 2.3. Increased Expression of Pax2 and GFAP in Transgenic Mice at P7

**Figure 3:**
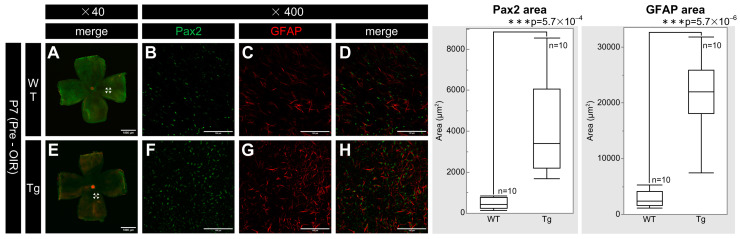
Confocal images of whole-mount retinas from P7 WT and PDGF-Atg mice immunostained for Pax2 (green) and GFAP (red) alongside a comparative box plot analysis. (**A**–**D**) Confocal images of WT retinas; (**B**–**D**) (Scale bar = 100 μm); enlarged images of the area outlined in (**A**) (scale bar = 1000 μm). (**E**–**H**) Images of PDGF-A transgenic mice; (**F**–**H**) scale bar = 100 μm; enlarged images of the area outlined in (**E**) (scale bar = 1000 μm). (**A**,**E**) Composite low-magnification images of staining for Pax2 and GFAP, respectively; (**B**,**F**) immunostaining with the Pax2 antibody; (**C**,**G**) immunostaining with the GFAP antibody; (**D**) is a combination of (**B**,**C**); and (**H**) is a combination of (**F**,**G**). In the retinas, Pax2 expression was increased in PDGF-A transgenic mice (**F**) compared to that in WT mice (**B**) (n = 10, *p* = 5.7 × 10^−4^ < 0.05). Similarly, GFAP expression was also increased in PDGF-A transgenic mice (**G**) compared to that in WT mice (**C**) (n = 10, *p* = 5.7 × 10^−6^ < 0.05). The corresponding box plot analysis, positioned adjacent to the images, illustrates the statistical comparisons. Retinal and statistical analyses were conducted as described in the Section 4, with n representing the number of mice.

PDGF-Atg mice had significantly higher expression of Pax2 and GFAP on the retinal surface than WT mice at P7, with retinal whole-mount preparations revealing enhanced staining for both markers in transgenic mice (Figure 3F–H) versus WT mice (Figure 3B–D). Quantitative analysis confirmed the statistically significant increases in fluorescence areas for both Pax2 (n = 10, *p* = 5.7 × 10^−4^) and GFAP (n = 10, *p* = 5.7 × 10^−6^) in transgenic mice compared to the WT controls. These results indicated a substantial upregulation of Pax2 and GFAP expression associated with PDGF-A overexpression.

### 2.4. Increased Expression of PDGF Receptor Alpha Isoform (PDGFRα) in Transgenic Mice at P7

**Figure 4:**
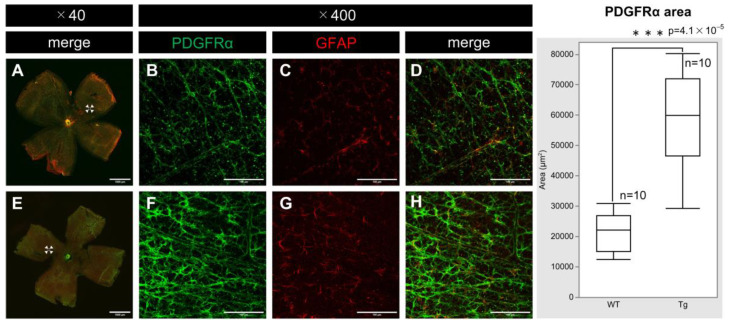
Confocal images of whole-mount of the retinas from P7 WT and PDGF-Atg mice immunostained for PDGFRα (green) and GFAP (red) antibody, alongside a comparative box plot analysis. (**A**–**D**) Confocal images of wild-type mice; (**B**–**D**) scale bar = 100 μm; enlarged images of the area outlined in (**A**) (scale bar = 1000 μm); (**E**–**H**) images of PDGF-A transgenic mice; (**F**–**H**) (scale bar = 100 μm); enlarged images of the area outlined in **e** (scale bar = 1000 μm); (**A**,**E**) are composite low-magnification images stained with PDGFRα and GFAP; (**B**,**F**) show immunostaining with PDGFRα antibody; (**C**,**G**) show immunostaining with GFAP antibody; and (**D**) is a merge of (**B**,**C**); while (**H**) is a merge of (**F**,**G**). In the retinas, PDGFRα expression was increased in PDGF-A transgenic mice (**F**) compared to that in WT mice (**B**) (n = 10, *p* = 4.1 × 10^−5^ < 0.05). The corresponding box plot analysis, positioned adjacent to the images, illustrates the statistical comparisons. Retinal and statistical analyses were conducted as described in the Methods section, with n representing the number of mice.

PDGF-Atg mice had significantly higher expression of PDGFRα compared to WT mice at P7. Confocal microscopy of whole-mounted retinas showed increased PDGFRα staining in transgenic mice (Figure 4F) relative to that in WT mice (Figure 4B). Moreover, this upregulation was particularly evident alongside the increase in the levels of GFAP (Figure 4C,G), suggesting enhanced PDGFRα expression within astrocytes. Quantitative analysis also demonstrated a substantial difference in PDGFRα expression between transgenic and WT mice (n = 10, *p* = 4.1 × 10^−5^), highlighting the impact of PDGF-A overexpression on PDGFRα levels.

### 2.5. Increased Vascular Density and Length in Transgenic Mice at P7

**Figure 5:**
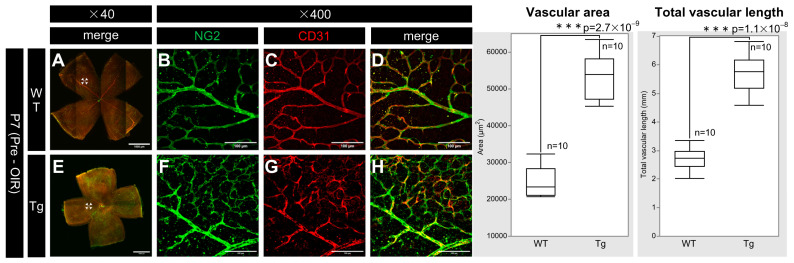
Confocal images of whole-mount of the retinas from P7 WT and PDGF-Atg mice immunostained with anti-NG2 (green) and anti-CD31 (red) antibody, alongside a comparative box plot analysis. (**A**–**D**) Confocal images of wild-type mice. (**B**–**D**) Scale bar = 100 μm; enlarged images of the area outlined in (**A**) (scale bar = 1000 μm). (**E**–**H**) Images of PDGF-A transgenic mice. (**F**–**H**) Scale bar = 100 μm; enlarged images of the area outlined in (**E**) (scale bar = 1000 μm). (**A**,**E**) show composite low-magnification images stained with NG2 and CD31, respectively; (**B**,**F**) show immunostaining with NG2 antibody; (**C**,**G**) show immunostaining with CD31 antibody; (**D**) is a combination of (**B**,**C**); and (**H**) is a combination of (**F**,**G**). In the retinas of PDGF-A transgenic mice, the expression of the vascular markers NG2 and CD31 was increased (**F**,**G**) compared to wild-type mice (**B**,**C**). Both CD31 and NG2 are components of the vascular structure, indicating increased vascular density (n = 10, *p* = 2.7 × 10^−9^ < 0.05) and length (n = 10, *p* = 1.1 × 10^−8^ < 0.05) in the retinas of the transgenic mice. The corresponding box plot analysis, positioned adjacent to the images, illustrates the statistical comparisons. Retinal and statistical analyses were conducted as described in the Methods section, with n representing the number of mice.

PDGF-Atg mice had higher retinal vascular density than WT mice at P7 (Figure 5B,C,F,G). Retinal whole-mount immunostaining for CD31 and NG2 (vascular markers for endothelial cells and pericytes, respectively) showed increased vascular density and length in transgenic mice, with significant increases in the fluorescence area (n = 10, *p* = 2.7 × 10^−9^) and total vascular length (n = 10, *p* = 1.1 × 10^−8^) for both markers in transgenic mice compared to that in WT mice.

### 2.6. Comparison of Vascular Regression Area After Hyperoxia Exposure in the OIR Model

**Figure 6:**
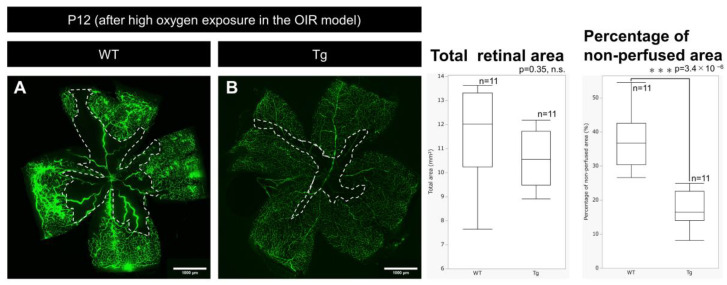
Images of whole-mount retinas taken after fluorescein perfusion from P12 (after high oxygen exposure) WT (**A**) and PDGF-Atg (**B**) mice (scale bar = 1000 μm) alongside comparative box plot analysis. The areas outlined by the white dashed lines indicate the regions of retinal vascular regression, which were analyzed automatically using ImageJ software (Fiji Ver. 1.54). Adjacent to the images, a table and box plot analysis were used to compare the total retinal area and non-perfused retinal area between the wild-type and PDGF-A transgenic mice. Whole-mount retinal fluorescein-staining shows a reduced vascular regression area after high oxygen exposure in PDGF-A transgenic mice compared to wild-type mice (n = 11, *p* = 3.4 × 10^−6^). Statistical analysis was performed as detailed in the Methods section, with n representing the number of mice analyzed.

PDGF-Atg mice showed significantly smaller areas of vascular regression immediately after hyperoxia exposure than WT mice at P12 after hyperoxic exposure, with fluorescein perfusion assays showing reduced retinal vascular regression in transgenic mice (Figure 6A,B; Table 1; n = 10, *p* = 3.4 × 10^−6^).

### 2.7. Altered Expression of Pericytes and Astrocytes Post-Hyperoxia Exposure

**Figure 7:**
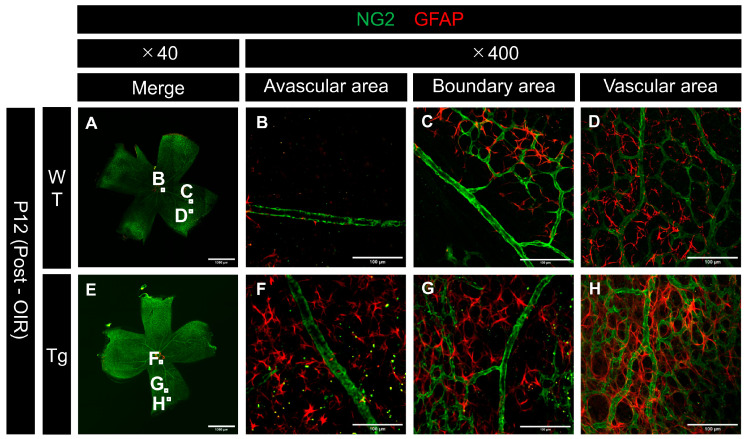
Confocal images of whole-mount retinas from P12 (after high oxygen exposure in the OIR model) WT and PDGF-Atg mice immunostained with anti-NG2 (green) and anti-GFAP (red) antibody. (**A–D**) Confocal images of wild-type mice; (**B**–**D**) scale bar = 100 μm; enlarged images of the area outlined in a (scale bar = 1000 μm); (**E**–**H**) images of PDGF-A transgenic mice; (**F**–**H**) scale bar = 100 μm; enlarged images of the area outlined in (**E**) (scale bar = 1000 μm); (**B**–**D**,**F**–**H**) images of a single major vein extending from the optic disc, capturing the non-perfused area (**B**,**F**), vascular area (**D**,**H**), and boundary area between them (**C**,**G**). Comparison of astrocyte (GFAP) expression in the non-perfused regions revealed low expression in wild-type mice (**B**) but relatively retained expression in PDGF-A transgenic mice (**F**). Similar trends were observed in the border regions (**C**,**G**). In the vascularized areas (**D**,**H**), both retained GFAP expression.

A notable difference was observed between the WT and PDGF-Atg mice in the comparison of astrocytes in P12 mouse retinas following hyperoxia exposure. GFAP expression was reduced in the avascular area around the main blood vessels in WT mice (Figure 7B), whereas this reduction was relatively absent in PDGF-Atg mice (Figure 7F). Examination of the boundaries between the vascular regression areas and the remaining vascular regions showed that GFAP expression tended to persist in the remaining vascular regions in WT mice (Figure 7C).

### 2.8. Differences in Vascular Structure Following Hyperoxic Exposure

**Figure 8:**
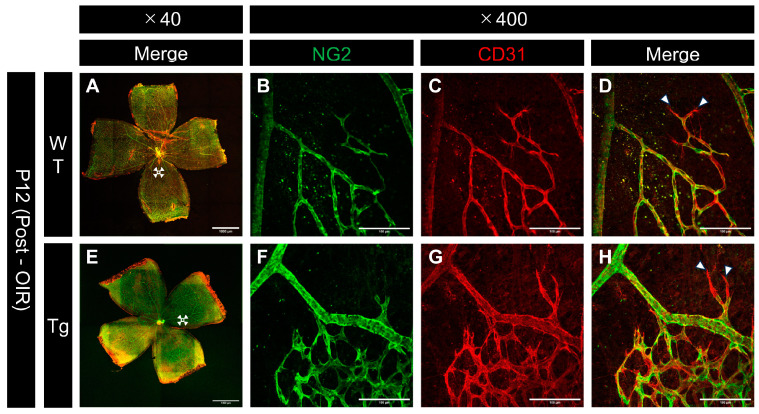
Confocal images of whole-mount retinas from P12 (after high oxygen exposure in the OIR model) WT and PDGF-Atg mice immunostained with anti-NG2 (green) and anti-CD31 (red) antibody. (**A**–**D**) Confocal images of wild-type mice; (**B**–**D**) scale bar = 100 μm; enlarged images of the area outlined in (**A**) (scale bar = 1000 μm); (**E**–**H**) images of PDGF-A transgenic mice; (**F**–**H**) scale bar = 100 μm; enlarged images of the area outlined in (**E**) (scale bar = 1000 μm); (**A**,**E**) composite low-magnification images stained with NG2 and CD31, respectively; (**B**,**F**) immunostaining with NG2 antibody; (**C**,**G**) immunostaining with CD31 antibody; (**D**) is a combination of (**B**,**C**); (**H**) is a combination of (**F**,**G**). The regions shown in (**B**,**C**,**F**,**G**) represent the boundary areas between the existing vasculature and the avascular areas. In both wild-type and transgenic mice, NG2 (pericyte marker) showed a greater degree of regression than CD31 (endothelial marker) at the edges of the regressing vessels (white arrowhead).

Confocal images of immunostained whole-mount retinas from P12 mice after hyperoxic exposure revealed differences in vascular regression patterns between WT and Tg mice. Assessment of both NG2 and CD31 at the edges of regressing vessels showed that pericytes tended to regress earlier than endothelial cells, particularly at the regressing vessel edges, in both WT and transgenic mice (Figure 8B,C,F,G). These observations suggest that pericytes are more susceptible to hyperoxia-induced damage than endothelial cells.

## 3. Discussion

### 3.1. PDGF-A Overexpression Enhanced Neurovascular Unit Stability

Astrocytes are integral components of the retinal neurovascular unit, supporting blood vessels by ensheathing them and secreting angiogenic factors such as VEGF [7]. The retinal vasculature essentially comprises CD31-positive endothelial cells enveloped by NG2-positive pericytes, and the integrity of this unit is crucial for maintaining the blood–retinal barrier (BRB), which in turn is essential for retinal homeostasis and function. The BRB consists of tightly regulated endothelial cells and pericytes as well as supporting astrocytes and prevents the entry of potentially harmful substances into the retinal tissue [12]. PDGF-A is essential for the recruitment and differentiation of astrocytes and thus is a critical contributor to the maintenance and stabilization of the neurovascular unit and the BRB [13].

Our study demonstrated that PDGF-Atg mice had significantly increased expression of GFAP, a major astrocytic protein, and Pax2, a marker of astrocyte precursor cells, indicating that PDGF-A promotes both the proliferation and maturation of astrocytes. Furthermore, PDGFRα expression was elevated in the transgenic mice, particularly in the regions rich in astrocytes. PDGFRα is a receptor for PDGF-A and is essential for signaling pathways that regulate astrocyte behavior, including proliferation, migration, and differentiation [13]. Thus, elevated PDGFRα expression underscores the specific targeting of astrocytes by PDGF-A signaling to enhance their stability and function. In addition to increased astrocyte expression, PDGF-Atg mice also had higher vascular density than WT mice. This suggests that PDGF-A overexpression promotes the formation of the entire neurovascular unit and not just astrocytes. The increase in vascular density also implies that PDGF-A fosters a conducive environment for endothelial cells and pericytes, leading to more stable and functional blood vessels. This comprehensive evidence underscores the therapeutic potential of PDGF-A in the treatment of retinal vascular diseases.

### 3.2. Inhibition of Avascular Area Formation After Hyperoxic Exposure

Under hyperoxic conditions, such as those encountered in ROP as well as in its experimental model of OIR, oxidative stress induces significant pathological changes in the retinal neurovascular unit. One of the critical effects of this stress is the retraction and dysfunction of astrocytes [14], ultimately resulting in a marked decrease in VEGF expression. The reduction in VEGF levels impairs the ability of blood vessels to survive and grow, leading to vascular regression characterized by the disintegration of endothelial cells and loss of pericyte support, ultimately resulting in compromised blood vessel integrity [15].

Our results strongly support the protective role of PDGF-A in retinal vascular stability under hyperoxic conditions. The area of vascular regression immediately after hyperoxia exposure was significantly smaller in PDGF-Atg mice than in WT mice, suggesting that PDGF-A exerts a protective effect that enhances astrocyte resilience and confers vascular protection.

Furthermore, GFAP expression in avascular areas in WT mice tended to decrease following hyperoxic exposure, indicating a loss of the astrocytic support critical for maintaining the integrity and function of the retinal vasculature. Conversely, in regions where the vasculature survived, astrocyte expression remained relatively stable, highlighting the dependence of astrocytes on a supportive vascular environment.

However, this scenario was notably different in PDGF-A transgenic mice. Astrocytes in avascular areas exhibit significantly less retraction after hyperoxic exposure, indicating that PDGF-A provides a protective effect that enhances astrocyte resilience. This preservation of astrocytic function correlated with a markedly smaller area of vascular regression compared to that in WT mice, underscoring the role of PDGF-A in enhancing the resistance of the neurovascular unit to hyperoxic conditions. This effect is crucial, as it suggests that PDGF-A stimulation can mitigate the adverse effects of hyperoxia, which are prevalent in conditions such as ROP.

### 3.3. Differential Regression of Pericytes and Endothelial Cells Under Hyperoxic Conditions

Further analysis revealed differential regression rates of pericytes and endothelial cells at the boundaries between vascular and avascular regions following hyperoxic exposure. In areas of vascular regression, pericytes were observed to retract first, followed by the subsequent and slower retraction of endothelial cells. This sequential regression indicates that the structural disintegration of blood vessels under hyperoxic conditions progresses inwards from the outer pericytes to the endothelial cells.

This regression pattern highlights the pivotal role of pericytes in maintaining vascular stability and their vulnerability to oxidative stress. Astrocytes and pericytes contribute to the maintenance of the tight junctions between endothelial cells that are crucial for BRB integrity. Under hyperoxic conditions, the retraction of astrocytes disrupts these tight junctions, making endothelial cells more susceptible to apoptosis and further compromising the BRB [12,14].

The increased expression of PDGFRα in astrocytes in PDGF-Atg mice suggests a direct mechanism by which PDGF-A signaling enhances astrocyte stability and functioning. It suggests that astrocytes may be the initial targets of the protective effects of PDGF-A during hyperoxic exposure, which subsequently cascade to preserve pericytes and endothelial cells. The proposed order of regression—from astrocytes to pericytes and then endothelial cells—reflects a hierarchical support system within the neurovascular unit, with astrocytes playing the most foundational role.

### 3.4. Therapeutic Implications for Retinal Vascular Diseases

These findings emphasize the potential of PDGF-A as a therapeutic agent for conditions characterized by oxidative stress and vascular instability. By enhancing astrocyte resilience and function, PDGF-A could contribute to the stabilization of the neurovascular unit, offering a promising strategy for mitigating diseases like ROP and diabetic retinopathy. Current treatments for ROP, including laser photocoagulation, anti-VEGF injections, and vitrectomy, primarily address the consequences of avascular area formation, but do not prevent its development [2]. Administering PDGF-A during hyperoxic exposure in premature infants could promote normal vascular development by recruiting astrocytes, potentially preventing avascular area formation and improving outcomes.

Notably, pericyte loss is a critical feature of diabetic retinopathy, contributing to BRB breakdown and subsequent vascular complications [16]. It is possible that prophylactic administration of PDGF-A could activate astrocytes and prevent avascular zone formation to promote the re-establishment of normal vascular structure in diabetic retinopathy. As it supports the entire neurovascular unit, PDGF-A could mitigate early vascular damage and prevent progression to more severe disease stages.

### 3.5. Limitations

#### 3.5.1. Considerations of Translational Relevance

The OIR mouse model demonstrates that high oxygen exposure leads to the regression of the retinal vasculature around the optic nerve head, creating avascular regions in this area. In contrast, high oxygen exposure results in poor vascular development and the formation of avascular zones as ROP in humans, primarily in the peripheral retina [4]. This discrepancy underscores the need for the careful translation of findings from mouse models to the clinical setting.

Overexpression of PDGF-A in the human retina could potentially lead to unintended consequences, particularly within the macula [17]. If PDGF-A induces vessel proliferation in the macular avascular zone, it may disrupt the delicate balance required for optimal visual acuity. Furthermore, an excessive increase in astrocyte numbers could interfere with the structural and functional integrity of the macula, potentially leading to vision impairment. Therefore, although PDGF-A treatment shows promise in stabilizing the retinal vasculature, its application in humans must be approached with caution. Future studies will be required to determine the specific effects of such treatments on macular health and to help develop strategies that target affected areas without compromising macular function.

#### 3.5.2. Limitations with Regard to GFAP as an Astrocyte Marker

There are also significant limitations associated with the use of GFAP as an astrocyte marker. Although GFAP is widely employed owing to its prevalent expression in astrocytes and was used in this study based on previous reports [11,12], its expression is not exclusive to astrocytes; GFAP can be upregulated in early response to neural injury, oxidative stress, inflammation, and retinal diseases not only in astrocytes, but also in Müller glial cells and other reactive glial cells [18]. This makes it challenging to accurately assess astrocyte populations and functions based solely on GFAP immunostaining. Nonetheless, other markers such as S100β, vimentin, Ki67, and ALDH1L1 also have limitations due to overlapping expression with other glial cells [19]. Furthermore, the studies of astrocyte markers have also been conducted in connective tissue models of rats in addition to mice, but differences in astrocyte morphology between species make direct applications or comparisons challenging [20]. Currently, no single marker can exclusively identify astrocytes without cross-reactivity, and methods to evaluate the functions of each glial cell type separately, especially in the complex retinal environment, are not fully established. Future research with more specific markers and advanced techniques will be needed to enable the precise evaluation of astrocytes and to enhance our understanding of the effects of interventions such as PDGF-A overexpression on retinal health.

### 3.6. Conclusions

PDGF-A overexpression in transgenic mice enhanced astrocyte proliferation and maturation, increased vascular density, and protected against hyperoxia-induced vascular regression. The differential regression of pericytes and endothelial cells under hyperoxic conditions highlights the hierarchical support within the neurovascular unit and emphasizes the foundational role of astrocytes. These effects also suggest that PDGF-A plays a crucial role in stabilizing the neurovascular unit and maintaining BRB integrity. However, although the potential therapeutic applications of PDGF-A in retinal vascular diseases are promising, careful consideration of its translational relevance and the potential risks is required. Further studies will be essential for elucidating the mechanisms of action of PDGF-A that could enable the development of safe and effective therapeutic strategies for human retinal diseases.

## 4. Materials and Methods

### 4.1. Experimental Animals

Transgenic mice were obtained from the Wilmer Eye Institute, Johns Hopkins University [10]. WT C57BL/6 mice were procured from CLEA Japan, Inc., Tokyo, Japan.

**Figure 9:**
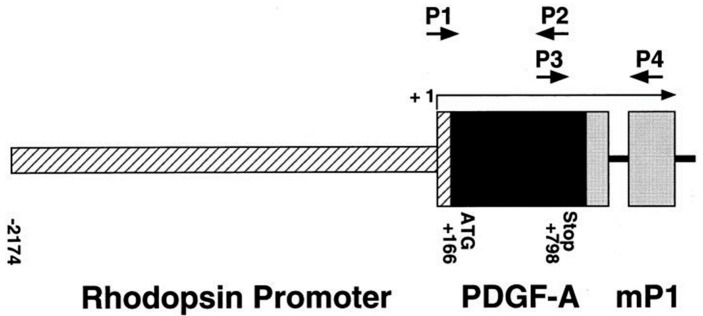
Schematic map of the rhodopsin/PDGF-A fusion gene. P1 and P2, oligonucleotide primers used to screen genomic DNA for the transgene; P3 and P4, primers used for RT-PCR; +1, transcription start site [10].

To prepare C57BL/6 background rho/PDGF-Atg mice, full-length complementary DNA (cDNA) for PDGF-AA was cloned into a plasmid with a 2.2 kb HindIII/NaeI fragment from the bovine rhodopsin promoter, an intron, a polyadenylic acid addition site derived from the mouse protamine gene, and a eukaryotic consensus ribosomal binding site. After transformation, a clone with the correct orientation was selected, and the DNA was double CsCl-purified and cleaved using EcoRI to yield the 3546 bp fusion gene (Figure 9).

To confirm the transgenic status, presumptive rho/PDGF-Atg mice were crossed with WT C57BL/6 mice, and the offspring litters were genotyped using PCR of tail DNA. Briefly, tail pieces were digested overnight at 55 °C in a solution containing 50 mM Tris (pH of 7.5), 100 mM EDTA, 400 mM NaCl, 0.5% SDS, and 0.6 g/L Proteinase K. PCR was performed at 58 °C using the primers P3 (5′-AACACGAGCAGTGTCAAGTGCCAG-3′) and P4 (5′-GATGTGGCGAGATGCTCTTGAAGTCTGGTA-3′), which amplify a 632 bp transgene-specific sequence. Transgenic parents were considered confirmed rho/PDGF-Atg mice if all offspring litters were transgene-positive.

These animals were housed in the Kansai Medical University animal experimentation facility within isolators at a temperature of 22 ± 3 °C and 60% humidity. The lighting conditions consisted of 12 h of dark (less than 10 lux) followed by 12 h of light (approximately 50 lux). To minimize and eliminate pain, mice were anesthetized with an intraperitoneal injection containing 0.3 mL/kg of Ceractar^®^ (Bayer, Berlin, Germany) and Ketalar^®^ (Daiichi Sankyo Co., Tokyo, Japan) (50 mg/mL ketamine and 5 mg/mL xylazine). Euthanasia was performed using the cervical dislocation method to ensure central destruction. This study which involved the use of mice was conducted in accordance with ethical standards and guidelines for animal experimentation and was duly approved by the Ethics Committee of Kansai Medical University (approval number 24-034).

### 4.2. OIR Model

Retinal vascular obliteration was induced using a well-established method [4]. Briefly, P7 mice were exposed to 75% oxygen for 5 days, followed by a return to room air for 5 days to mimic the fluctuating oxygen levels encountered during ROP. Typically, proliferative neovascularization in this model is assessed on P17; however, as we focused on the vascular regression occurring during high oxygen exposure in this study, the mice were sacrificed on P12, i.e., 5 days after high oxygen exposure, to obtain samples for specifically examining the effects of hyperoxia on vascular obliteration.

### 4.3. Sample Collection

Mice were euthanized by cervical dislocation, a method approved by our institutional animal care and use committee, and the eyeballs were carefully enucleated immediately to avoid retinal damage.

### 4.4. Western Blotting

Retinal tissues were collected and homogenized in ice-cold UltraRIPA^®^ Kit for Lipid Raft (BioDynamics Laboratory, Tokyo, Japan) solution supplemented with a protease inhibitor cocktail (Roche, Basel, Switzerland) to prepare total protein lysates. The homogenates were centrifuged at 20,000× *g* for 15 min at 4 °C to remove debris and obtain the supernatant. Protein concentration in the supernatant samples was determined using a BCA kit (Bio-Rad, Hercules, CA, USA). Thereafter, equal amounts of protein (20 μg) from each sample were mixed with Laemmli buffer (Bio-Rad, Hercules, CA, USA) containing β-mercaptoethanol (FUJIFILM Wako Pure Chemical Corporation, Osaka, Japan) and boiled at 95 °C for 5 min. The samples were separated by SDS-PAGE on a 10% polyacrylamide gel and transferred onto a PVDF membrane using the Trans-Blot Turbo Transfer System (Bio-Rad, Hercules, CA, USA) according to the manufacturer’s instructions.

Membranes were blocked using EveryBlot Blocking Buffer (Bio-Rad, Hercules, CA, USA) for 1 h at room temperature. After blocking, the membranes were incubated overnight at 4 °C with primary antibodies specific to the target proteins diluted in the same blocking buffer. The primary antibodies used in this study are as follows: anti-PDGF-A (1:1000; Santa Cruz Biotechnology, Dallas, TX, USA; Cat# sc-9974; mouse monoclonal) and anti-β-actin (1:3000; Cell Signaling Technology, Danvers, MA, USA; Cat# 4970S; rabbit monoclonal). β-actin was used as the loading control. Following primary antibody incubation, the membranes were washed three times in TBS-T and then incubated with appropriate horseradish peroxidase-conjugated secondary antibodies (1:10,000; Jackson ImmunoResearch, West Grove, PA, USA) for 1 h at room temperature. The membranes were then washed again and developed using the ImmunoStar LD chemiluminescence detection kit (FUJIFILM Wako Pure Chemical Corporation, Osaka, Japan). Protein bands were visualized using a Fusion Solo imaging system (Vilber Lourmat, Collégien, France). Densitometric analysis was performed using ImageJ (NIH, Bethesda, MD, USA), with protein expression levels normalized to that of β-actin.

### 4.5. Immunofluorescence Analysis of Whole-Mount Retinas

After enucleation, eyeballs were immersed in 4% paraformaldehyde for 1 h. The cornea and the lens were carefully removed to expose the retina, which was gently separated from the underlying retinal pigment epithelium using fine microsurgical tools. To ensure optimal preservation of retinal architecture, the isolated retina was re-fixed in 4% paraformaldehyde overnight at 4 °C.

Immunofluorescent staining of the retinas was performed using the following primary antibodies: anti-Pax2 (1:500; Abcam, Cambridge, UK; Cat# ab79389, rabbit monoclonal), anti-GFAP (1:500; Abcam, Cambridge, UK; Cat# ab279291, rat monoclonal), anti-CD31 (1:500; BD Pharmingen™, San Jose, CA, USA; Cat# 550274, rat monoclonal), anti-NG2 (1:500; Abcam, Cambridge, UK; Cat# ab259324, rabbit polyclonal), and anti-PDGFRα (1:500; Abcam, Cambridge, UK; Cat# ab203491, rabbit polyclonal). The cells were then incubated overnight at 4 °C. Following primary antibody incubation, the retinas were treated with secondary antibodies conjugated with Alexa Fluor^®^ 488 and Alexa Fluor^®^ 546 (1:800, Life Technologies, Carlsbad, CA, USA) for 3 h at room temperature. Secondary antibodies were selected based on the host species of primary antibodies. During the secondary antibody incubation, simultaneous 4′,6-diamidino-2-phenylindole (DAPI) staining was performed to label the cell nuclei.

After antibody incubation, the retinas were washed three times in PBS for 30 min each with gentle rocking. The retinas were carefully spread on a glass slide to ensure that they lay flat, with the photoreceptor side facing downward. Finally, the retinas were whole-mounted in ProLong™ Gold Antifade Mountant (Thermo Fisher Scientific, Waltham, MA, USA).

### 4.6. Immunofluorescence Analysis of Frozen Retinal Sections

To prepare the frozen retinal sections, eyeballs from the mice were immediately embedded in OCT compound (Sakura, Tokyo, Japan) for cryopreservation. During the embedding process, ink marks were made on the eyeballs to ensure clear identification of the superior and inferior poles. The embedded eyeballs were sectioned into 8 μm thick slices using a cryostat (Leica Biosystems, Wetzlar, Germany), and the sections were carefully placed on MAS-coated glass slides (Matsunami Glass Ind., Ltd., Kishiwada, Japan). For the immunostaining for PDGF-A and vascular structure, the sections were incubated with primary antibodies, including anti-PDGF-A (1:1000; Santa Cruz Biotechnology, Dallas, TX, USA, Cat# sc-9974; mouse monoclonal) and anti-CD31 (1:500; BD Pharmingen™, San Jose, CA, USA; Cat# 550274; rat monoclonal) overnight at 4 °C. After primary antibody incubation, the sections were treated with secondary antibodies (Alexa Fluor^®^ 488-conjugated; 1:800 dilution; Life Technologies, Carlsbad, CA, USA) for 1 h at room temperature. During the secondary antibody incubation, DAPI staining (Cellstain DAPI Solution; FUJIFILM Wako Pure Chemical Corporation, Osaka, Japan) was performed simultaneously to label the cell nuclei. Following the staining process, the sections were mounted using ProLong Gold Antifade Mountant (Thermo Fisher Scientific, Waltham, MA, USA).

### 4.7. Fluorescein Visualization of Retinal Vasculature

Mice were anesthetized with an intraperitoneal injection of a mixture of 0.3 mL/kg of Ceractar^®^ (Bayer, Berlin, Germany) and Ketalar^®^ (Daiichi Sankyo Co., Tokyo, Japan) (50 mg/mL ketamine and 5 mg/mL xylazine). Following deep anesthesia, fluorescein isothiocyanate-dextran (FITC-Dextran, Sigma-Aldrich, St. Louis, MO, USA; average molecular weight, 4000) was administered via cardiac perfusion using a syringe after thoracotomy to expose the heart. This procedure ensured the thorough circulation of fluorescein throughout the body. The eyes were enucleated after perfusion, the enucleated eyeballs were immersed in 4% paraformaldehyde for 1 h, and the cornea and the lens were carefully removed to expose the retina. The retina was gently separated from the underlying retinal pigment epithelium using fine microsurgical tools, and the isolated retina was then re-fixed in 4% paraformaldehyde overnight at 4 °C to ensure optimal preservation of the retinal architecture. Thereafter, the retinas were flat-mounted on glass slides with the photoreceptor side facing down and covered with ProLong Gold Antifade Mountant (Thermo Fisher Scientific, Waltham, MA, USA).

### 4.8. Image Acquisition and Analysis

The FV3000 microscope (Olympus, Tokyo, Japan) was used for confocal microscopy. Image acquisition settings, including exposure time, gain, and pinhole size, were standardized across all samples to ensure the comparability and reproducibility of the results. Care was taken to avoid overexposure or underexposure as a possible reason for loss of detail or increased background noise, respectively.

Image analysis was performed using ImageJ (NIH, Bethesda, MD, USA). For each image, regions of interest (ROIs) were defined, and fluorescence intensity within these areas was automatically measured using the software.

Total vascular length was measured using AngioTool [21] following the protocol outlined for vascular network quantification.

### 4.9. Statistical Analysis

To compare PDGF-A expression using Western blotting analysis, three separate mice from each group (WT and PDGF-Atg) were analyzed using the aforementioned methods.

Additionally, the total GFAP, Pax2, and PDGFRα expression areas as well as the total vascular area and total vascular length were compared. Each group had 10 mice, and one retina was collected from each mouse. Retinal measurements were obtained from 10 randomly selected peripheral regions, and the average of these measurements was used for the analysis.

Eleven mice were used in each group to compare ischemic areas after OIR. One retina was collected from each mouse and analyzed. Therefore, “n” in the figures and tables represents the number of mice.

Since this study does not involve observing temporal changes, no specific criteria were established for excluding animals or data points. However, in cases where fundamental experimental conditions were not met, such as failure to properly regulate oxygen concentration, data exclusion was considered. No data were ultimately excluded.

To evaluate the differences between groups, independent two-sample *t*-tests (two-tailed) were performed for each parameter. Welch’s *t*-test was used to account for differences in variance. Statistical significance was set at *p* < 0.05, and all analyses were performed using JMP^®^ (SAS Institute, Cary, NC, USA).

## Figures and Tables

**Table 1:** Total retinal area and non-perfused area in P12 WT and PDGF-A transgenic mice after high oxygen exposure in the OIR model.

	WT (n = 11)	Tg (n = 11)
Total retinal area (mm^2^)	11.39 ± 1.96	10.74 ± 1.11
Non-perfused area (mm^2^)	4.16 ± 0.93	1.80 ± 0.52
Percentage of non-perfused area (mm^2^)	37.10 ± 8.34	16.97 ± 5.30

The percentage of non-perfused area was calculated as a proportion of the total retinal area.

## Data Availability

All data generated or analyzed during this study are available from the corresponding author upon reasonable request.
